# Identification of a novel immune microenvironment signature predicting survival and therapeutic options for bladder cancer

**DOI:** 10.18632/aging.202327

**Published:** 2020-12-19

**Authors:** Yilin Yan, Zhengnan Huang, Jinming Cai, Pengfei Tang, Fang Zhang, Mingyue Tan, Bing Shen

**Affiliations:** 1 Department of Urology, Shanghai General Hospital, Shanghai Jiaotong University School of Medicine, Shanghai 200080, China; 2 Department of Urology, Shuguang Hospital, Shanghai University of Traditional Chinese Medicine, Shanghai 200021, China

**Keywords:** bladder cancer, immune signature, glycolysis, immune checkpoint inhibitor

## Abstract

Few studies have investigated the potential of tumor immune microenvironment genes as indicators of urinary bladder cancer. Here, we sought to establish an immune-related gene signature for determining prognosis and treatment options. We developed a ten-gene tumor immune microenvironment signature and evaluated its prognostic capacity on internal and external cohorts. Multivariate Cox regression and nomogram analyses revealed the prognostic risk model as an independent and effective indicator of prognosis. We observed lower proportions of CD8+ T cells, dendritic cells, regulatory T cells, higher proportions of macrophages and neutrophils in high UBC risk group. UBC tissues with high-risk score tend to exhibit high TP53 and RB1 mutation rates, high PD1/PD-L1 expression and poor-survival basal squamous subtypes, while those with low-risk score tend to have high FGFR3 mutation rates and luminal papillary subtypes. Unexpectedly, we found a highly significant positive correlation between glycolytic genes and risk score, highlighting metabolic competition in tumor ecosystem and potential therapeutic avenues. Our study thus revealed a tumor immune microenvironment signature for predicting prognostic and response to immune checkpoint inhibitors against bladder cancer. Prospective studies are required to further test the predictive capacity of this model.

## INTRODUCTION

Globally, urinary bladder cancer (UBC) is one of the most common malignancies of the urinary tract, with an estimated 550,000 new cases and 200,000 deaths [1]. UBC is generally subdivided into 2 classes, non-muscle invasive bladder cancer (NMIBC), which accounts for about 75% of UBC cases, or muscle-invasive bladder cancer (MIBC) that makes up about 25% of the cases. NMIBC is often treatable but is associated with frequent relapse. Up to 15% of NMIBC cases progress into MIBC, which is more aggressive [2]. Despite recent advances in cancer diagnosis and treatment, UBC is still associated with a high rate of metastasis [3]. Thus, there is an urgent need to better understand the molecular basis of UBC development and progression so as to improve outcomes.

Currently the standard treatment of MIBC is cisplatin-based chemotherapy [4]. Recent studies have demonstrated that UBC is characterized by high heterogeneity and genomic instability [5, 6]. UBC resulting from various driver events responds differently to treatment. For example, tumors characterized by a mesenchymal related signature appear more sensitive to immune checkpoint inhibitors and resistant to cisplatin-based chemotherapy [7].

Cancer immunotherapy has shown promise against various cancers [8–10]. Immune checkpoint inhibitor-based immunotherapy targeting programmed cell with cytotoxic T lymphocyte antigen 4 (CTLA4), programmed cell death 1 (PD1) and death-ligand 1 (PD-L1) have shown significant efficacy against advanced cancer [11, 12]. While UBC has been reported to respond to immune checkpoint inhibitors, the response to the outcomes have been poor [13]. Despite low efficacy, pembrolizumab (anti-PD1) is approved for treating metastatic UBC [14]. The tumor immune microenvironment (TME) is thought to influence UBC progression and understanding its characteristics may unveil biomarkers for immunotherapy effectiveness and precision treatment.

However, from the immunotherapy perspective, recent studies have discussed the role of tumor glycolysis in therapeutic resistance [15]. Enhancing glycolysis has been reported to be utilized and manipulated by bladder tumor cells to promote tumor progression [16]. The glycolytic switch of cancer cell, namely “aerobic glycolysis” or “Warburg effect”, not only promotes tumor growth and metastasis but also contributes to the acidic microenvironment by releasing lactate into the extracellular environment, limiting the development of an effective antitumor immune response. Sporadic reports have demonstrated that inhibition of tumor glycolysis could promote antitumor immune response [17]. Thus, glycolysis blockade in cancer cells could provide a novel approach to overcoming immunotherapy resistance.

Data-mining the deposited information of multi-omics data using bioinformatic methods have become essential tools for a deeper understanding of tumor biology [18, 19]. Transcriptome profiling has uncovered various gene signatures that may predict UBC risk [20, 21]. Multiple immune-related genes (IRGs) risk models have been proposed for diagnosis and prognosis of various cancers, including colorectal cancer [22], melanoma [23] and clear-cell renal cell cancer [24]. However, most studies have focused on diagnostic gene signatures for UBC [25, 26]. Therefore, there is an urgent need to develop a modified prognostic risk model of UBC to predict outcome and treatment options.

Herein, we evaluated publicly available UBC datasets, aiming to identify an IRG signature associated UBC prognosis (Supplementary Figure 1). We identified 158 differentially expressed IRGs in UBC samples and constructed an IRG risk model using Cox and Lasso regression analyses. We then validated the IRG signature by evaluating its prognostic accuracy and the relationship of the signature with clinicopathological features. Changes in tumor immune cell infiltration, mutation profile, molecular subtypes and glycolytic biological pathway associated with the risk model of UBC were also explored. Finally, potential upstream regulatory transcription factors for the ten genes in the signature were analyzed. Our study may help provide a deeper understanding and more specific personalized therapies for UBCs.

## RESULTS

### Identification of differentially expressed IRGs in BLCA tissues

**Figure 1:**
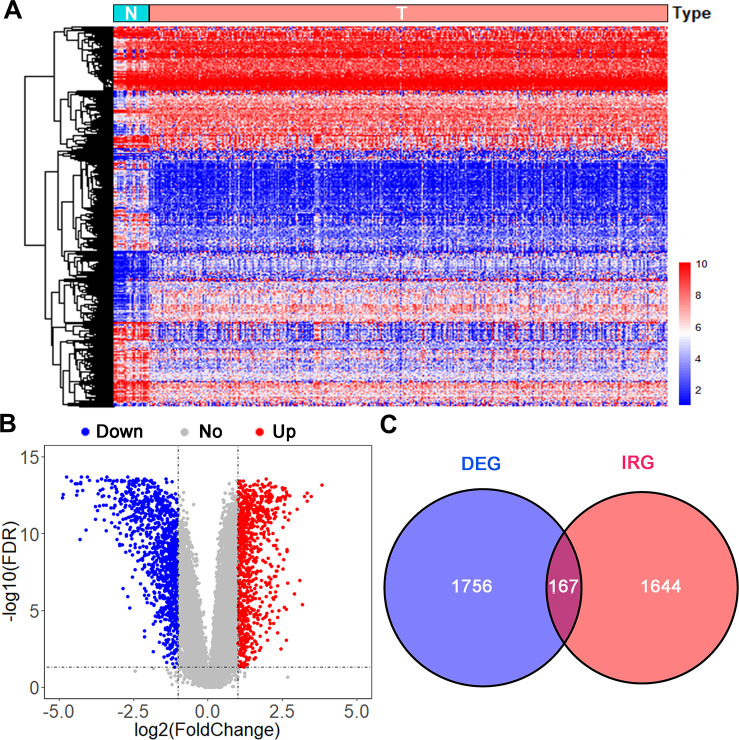
**Analysis of differentially expressed immune-related genes.** (**A**) Heatmap and (**B**) volcano plot of differentially expressed genes (DEGs) in UBCs compared with normal healthy bladder samples in datasets from TCGA and GTEx. (**C**) Venn diagram of the 158 intersecting genes between DEGs and IRGs in UBCs.

Data on 403 UBC cases were downloaded from TCGA (TCGA-BLCA dataset) and randomly split into a training group (n=202) and the testing group (n=201). Another independent UBC dataset (GSE13507) downloaded from GEO constituted an independent testing set (n=165). Detailed demographics and clinicopathologic characteristics of patients are listed in Table 1. After applying cutoff thresholds of |log2FC|>1 and FDR<0.05, 1,924 differentially expressed genes between normal urothelium tissues and bladder tumors from TCGA and GTEx were identified using the Wilcoxon signed-rank test. Of these, 870 were upregulated and 1,054 downregulated (Figure 1A, 1B). 158 differentially expressed IRGs (DEIRGS) were extracted from these gene lists (Figure 1C).

**Table 1:** Clinical data in the training and testing sets.

**Variables**	**Group**	**TCGA-BLCA**			**GSE13507 Testing cohort (n = 152)**
		**Total cohort (n = 403)**	**Training cohort (n = 202)**	**Testing cohort (n =201)**	
OS (months)					
	Mean	25.5	24.0	27.1	48.38
	Range	0.4-168.3	0.4-168.3	0.6-168.0	1.03-136.97
Vital status					
	Living	248	127	121	96
	Dead	155	75	80	69
T stage					
	Ta/1	4	2	2	104
	T2	117	59	58	31
	T3	192	95	97	19
	T4	57	28	29	11
	Unknown	33	18	15	
N stage					
	Negative	235	123	112	149
	Positive	126	61	65	15
	Unknown	42	18	24	1
M stage					
	M0	195	99	96	158
	M1	11	5	6	7
	Unknown	197	98	99	0
Grade					
	Low	20	10	10	105
	High	380	190	190	60
	Unknown	3	2	1	0
Age (years)					
	Mean	67.9	68.1	67.7	65.2
	Range	34-89	37-88	34-89	24-88
Gender					
	Male	298	146	152	135
	Female	105	56	49	30

### Functional enrichment analyses of DEIRGS

**Figure 2:**
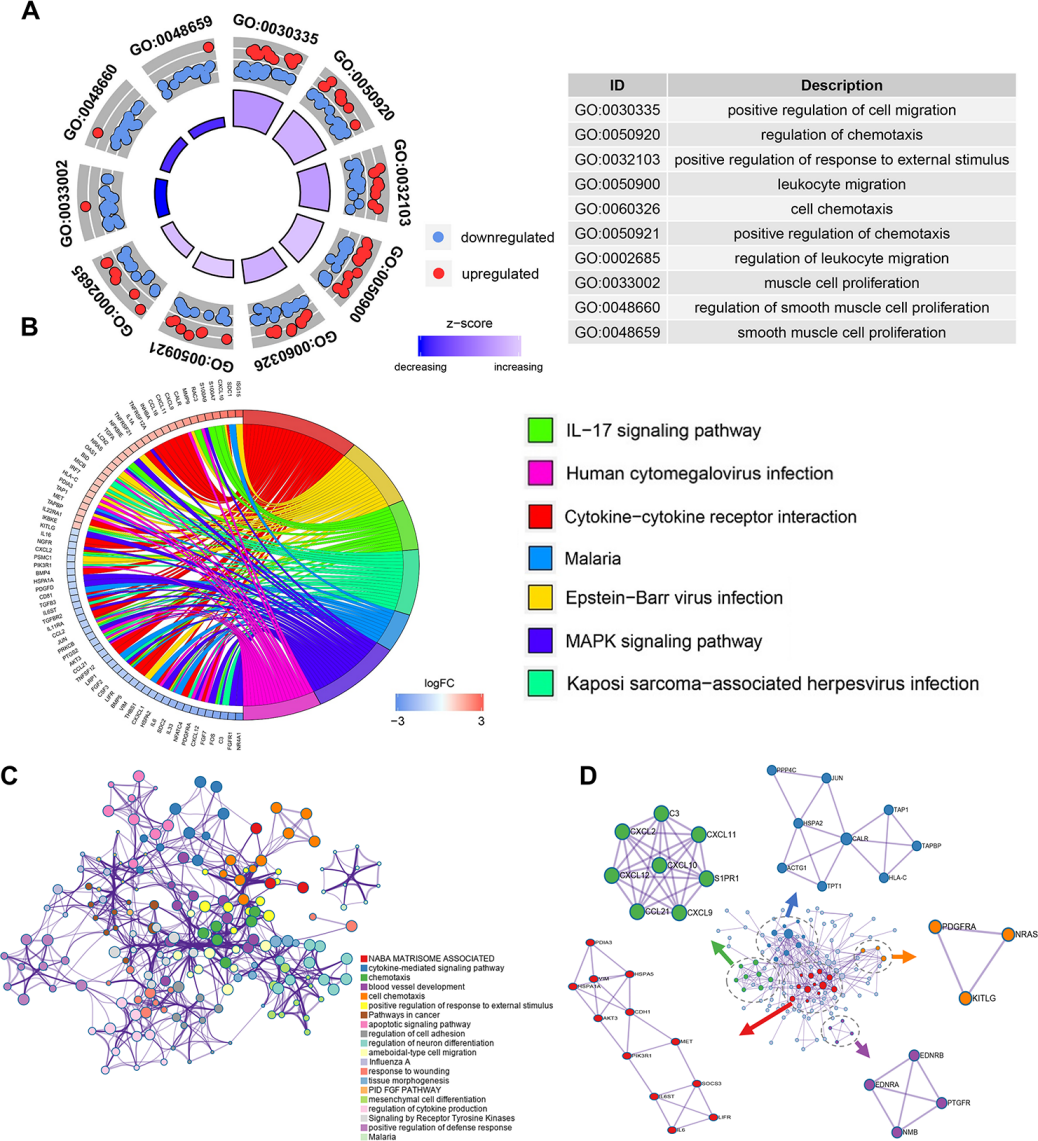
**Enrichment and protein-protein interaction analysis of differentially expressed IRGs.** (**A**) GO analysis of the immune-related genes. (**B**) Circular plot of the top seven most significant KEGG pathways. (**C**) Network plot of enriched terms: colored by cluster ID, where terms with a similarity > 0.3 are connected by edges. (**D**) Protein-protein interaction network and MCODE components identified in the gene lists.

To explore the potential function of the DEIRGS, a functional enrichment analysis was performed. Gene Ontology (GO) terms enrichment analysis was done and the 10 most enriched terms for biological process (BP), cellular component (CC), and molecular function (MF) identified (Figure 2A). Among them, “positive regulation of cell migration”, “regulation of chemotaxis” and “cell proliferation” were considered related to cancer progression. Kyoto Encyclopedia of Gene and Genomes (KEGG) analysis identified the 7 pathways enriched for in the DEIRGS, such as “IL-17 signaling pathways”, “cytokine-cytokine receptor interaction”, and “MAPK signaling pathway” (Figure 2B). To further illustrate the relationships between the enriched terms, terms of 158 DEIRGs were extracted and presented as a network plot (Figure 2C). Protein-protein interaction enrichment analysis identified 6 closely connected network components (Figure 2D).

### Construction of a 10-gene risk signature using the UBC training group

To explore the prognostic value of the above 158 genes, we performed univariate Cox regression analysis in the UBC training group and identified 14 DEGs as significantly associated with UBC survival (*p*<0.05). Analysis of the 14 DEIRGs using LASSO to minimize overfitting (Supplementary Figure 2A, 2B) identified 10 IRGs as risk genes (Table 2). The 10 genes were then used to construct an immune-related model using the following formula: risk score = 0.2427 * AHNAK + (0.3584 * CALR) + (-0.0863 * PLA2G2A) + (-0.1880 * OAS1) + (-0.0401 * CDH1) + (0.2079 * PDGFRA) +

**Table 2:** Coefficients and LASSO Cox model results of each gene in 10-IRG risk signature.

**Gene**	**HR**	**95% CI**	**p value**	**LASSO regression coefficient**
*AHNAK*	1.372	1.083-1.739	0.008	0.243
*CALR*	1.82	1.169-2.832	0.008	0.358
*PLA2G2A*	0.902	0.816-0.996	0.043	-0.086
*OAS1*	0.726	0.583-0.904	0.004	-0.188
*CDH1*	0.851	0.730-0.991	0.038	-0.040
*PDGFRA*	1.348	1.087-1.672	0.006	0.208
*SEMA3F*	0.811	0.671-0.981	0.031	-0.001
*RAC3*	1.336	1.077-1.657	0.008	0.228
*IRF5*	0.702	0.545-0.905	0.006	-0.070
*CARD11*	0.745	0.632-0.879	0.0005	-0.119

CI, confidence interval; HR, hazard ratio.

(-0.0014 * *SEMA3F*) + (0.2281 * *RAC3*) + (-0.0702 * *IRF5*) + (-0.1192 * *CARD11*).

**Figure 3:**
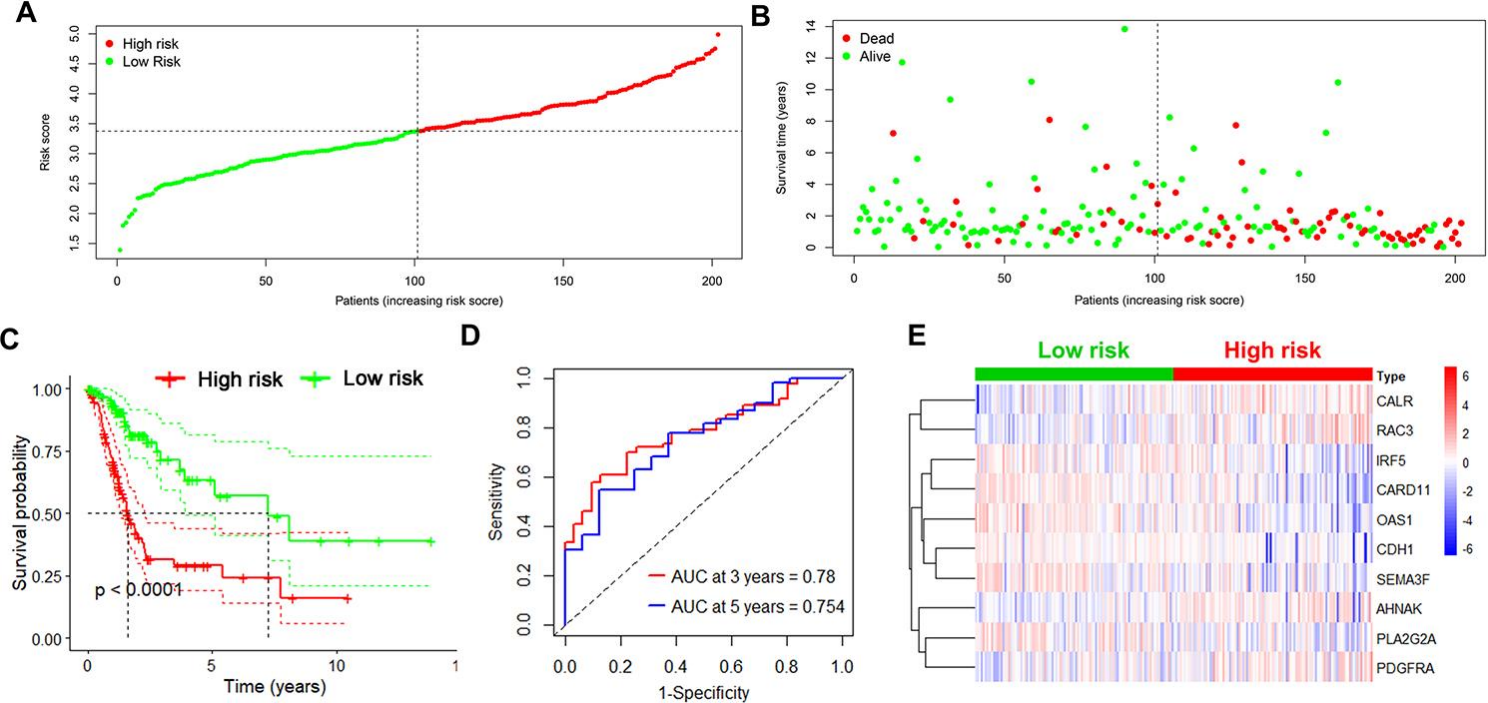
**Identification of the 10-IRG risk model in the training set.** (**A**, **B**) Risk score distribution and survival status of low-risk (green) (n=101) and high risk (red) UBC cases (n=101) in the training group. (**C**) Survival analysis of the high-risk UBC (n=101) and low-risk UBC (n=101) cases. (**D**) Time-dependent ROC curve analysis showing the 3-year (red) and 5-year (blue) survival of the 10 IRGs expression patterns. (**E**) Heat map showing expression level of risk genes in the high and low-risk BLCA patients in the training set.

Next, the risk score in each UBC case was calculated and ranked based on its expression of the 10 risk genes. The UBC cases in the training cohort were divided into a high-risk group (n =101) and a low-risk group (n=101) based on the median risk score (Figure 3A). The survival status of the UBC cases was visualized on dot plots (Figure 3B). Kaplan-Meier analysis revealed significantly different survival rates between the two groups (p<0.05; Figure 3C). The AUC value for the risk model was 0.780 at 3 years and 0.754 at 5years for overall survival (OS; Figure 3D). Expression levels of the risk genes in the 2 groups were visualized by heatmaps (Figure 3E).

### Internal and external validation of the 10-gene risk model

**Figure 4:**
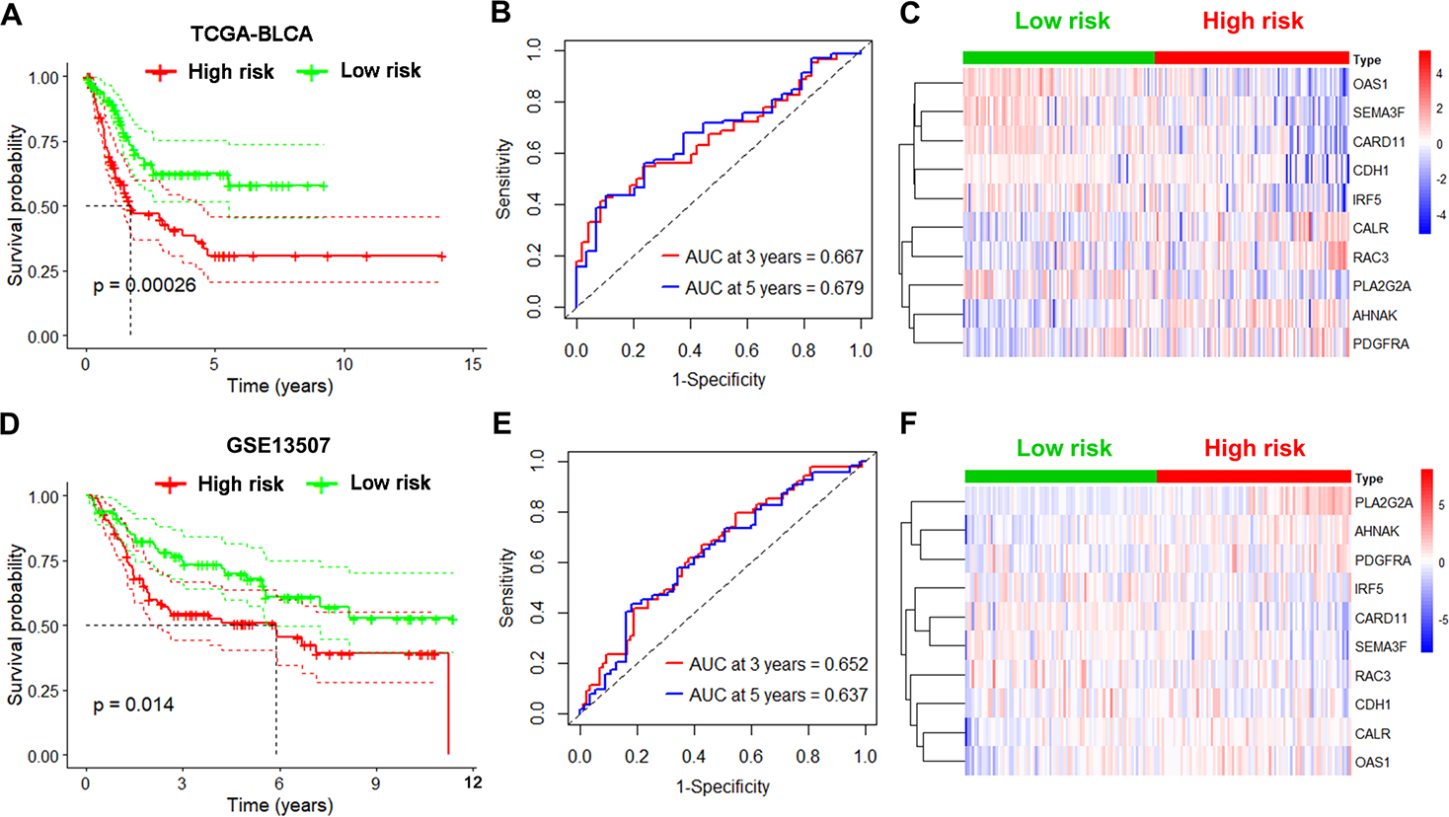
**Validation of the immune-based risk model in the internal TCGA and external GEO testing cohorts.** (**A**, **D**) Kaplan-Meier curve analysis of overall survival of high-risk UBC (n=101 for TCGA; n=83 for GSE13507) and low-risk UBC (n=100 for TCGA; n=82 for GSE13507) cases. (**B**, **E**) Time-dependent ROC curve analysis showing the 3-year and 5-year survival of the10 IRGs signature. (**C**, **F**) Heatmaps showing expression of the selected genes in the immune-based risk model.

Next, we tested the robustness of the 10-gene signature on 2 independent datasets. First, we repeated the analysis as described above in the internal TCGA testing group and observed lower survival rates for high-risk UBC cases relative to low-risk ones (p<0.05; Figure 4A). ROC curve analysis of overall survival revealed that the value of the AUC was 0.667 for 3 years and 0.679 for 5 years (Figure 4B). Risk genes expression levels in the internal testing group were visualized on heatmap (Figure 4C)**.** Next, we validated the 10-gene risk model in the external GEO testing dataset. The cases were first grouped into a low-risk group (n=82) and a high-risk group (n=83) based on median risk scores. Interestingly, cases with high-risk had shorter median survival relative to low-risk ones (p<0.05; Figure 4D). In the GEO dataset, the AUC values were 0.652 at 3 years and 0.637 at 5 years (Figure 4E). The expression of risk genes in the GEO testing cohort were visualized in heatmap (Figure 4F). These data indicate that the immune-related risk model accurately predicts UBC prognosis.

### Relationship between the 10-gene risk model and clinicopathological features

**Figure 5:**
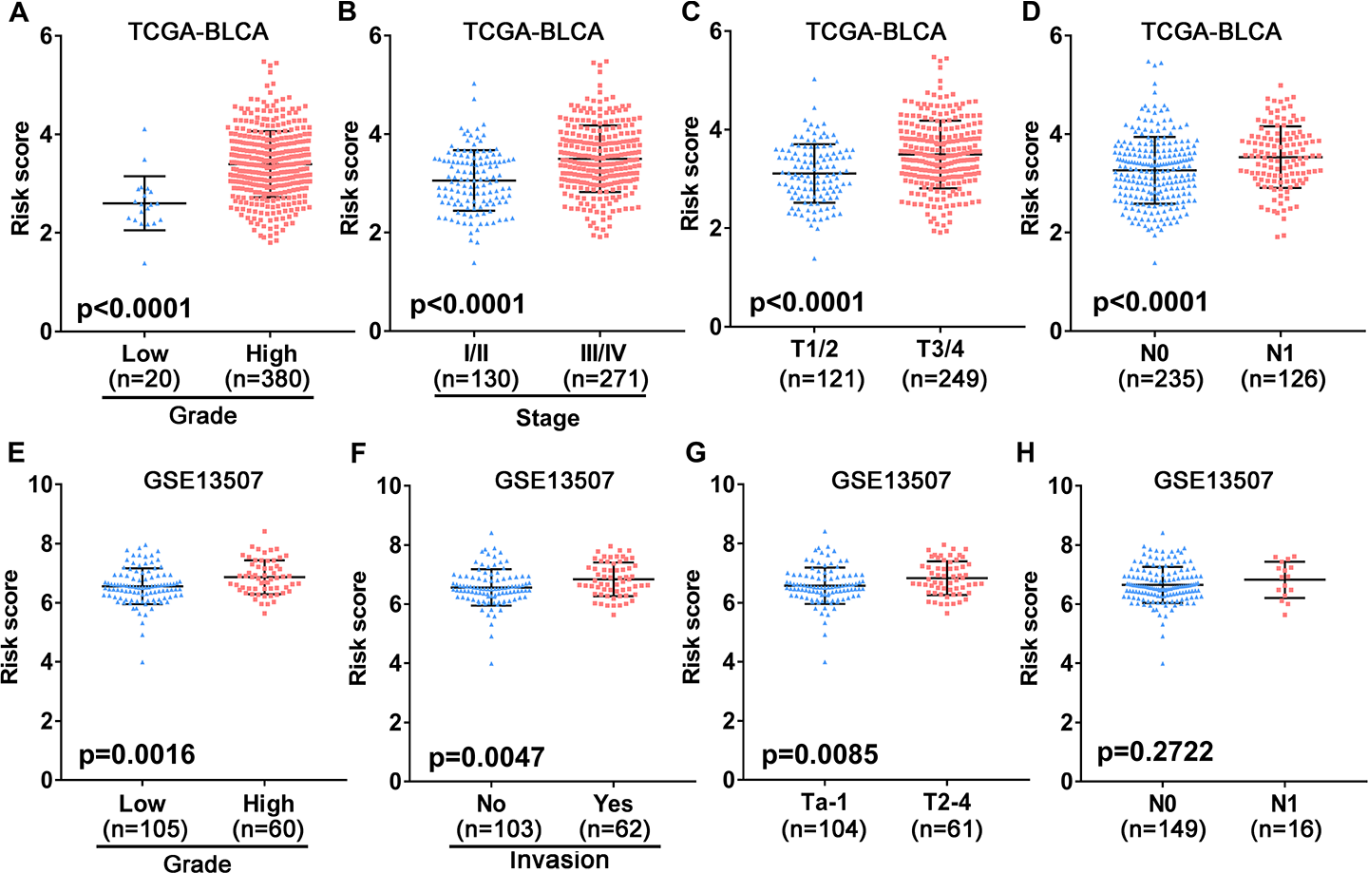
**Association between the 10-gene risk model and clinicopathological factors.** (**A**–**D**) Correlation of the risk score and clinicopathological factors including (**A**) grade, (**B**) clinical stage, (**C**) T stage, (**D**) N stage in the TCGA-BLCA patient cohort. (**E**–**H**) Association between the risk score and clinicopathological features including (**E**) histological grade, (**F**) invasive status, (**G**) T stage and (**H**) N stage in the GEO UBC patient cohort.

To assess the clinical value of the immune gene-related signature, we evaluated the association between the 10-gene risk model and UBC clinicopathological features. This analysis showed that signature risk score positively correlates with grade, clinical stage, pathological T stage and metastatic lymphatic status (Figure 5A–5D). In the independent GEO cohort, the 10-IRG risk score was significantly elevated in high grade and high muscle-invasive UBC cases relative to low grade and superficial UBCs (Figure 5E–5G). No difference was observed between risk score and N stage (Figure 5H). These data suggested that our IRG risk model closely correlates with clinical UBC features.

**Figure 6:**
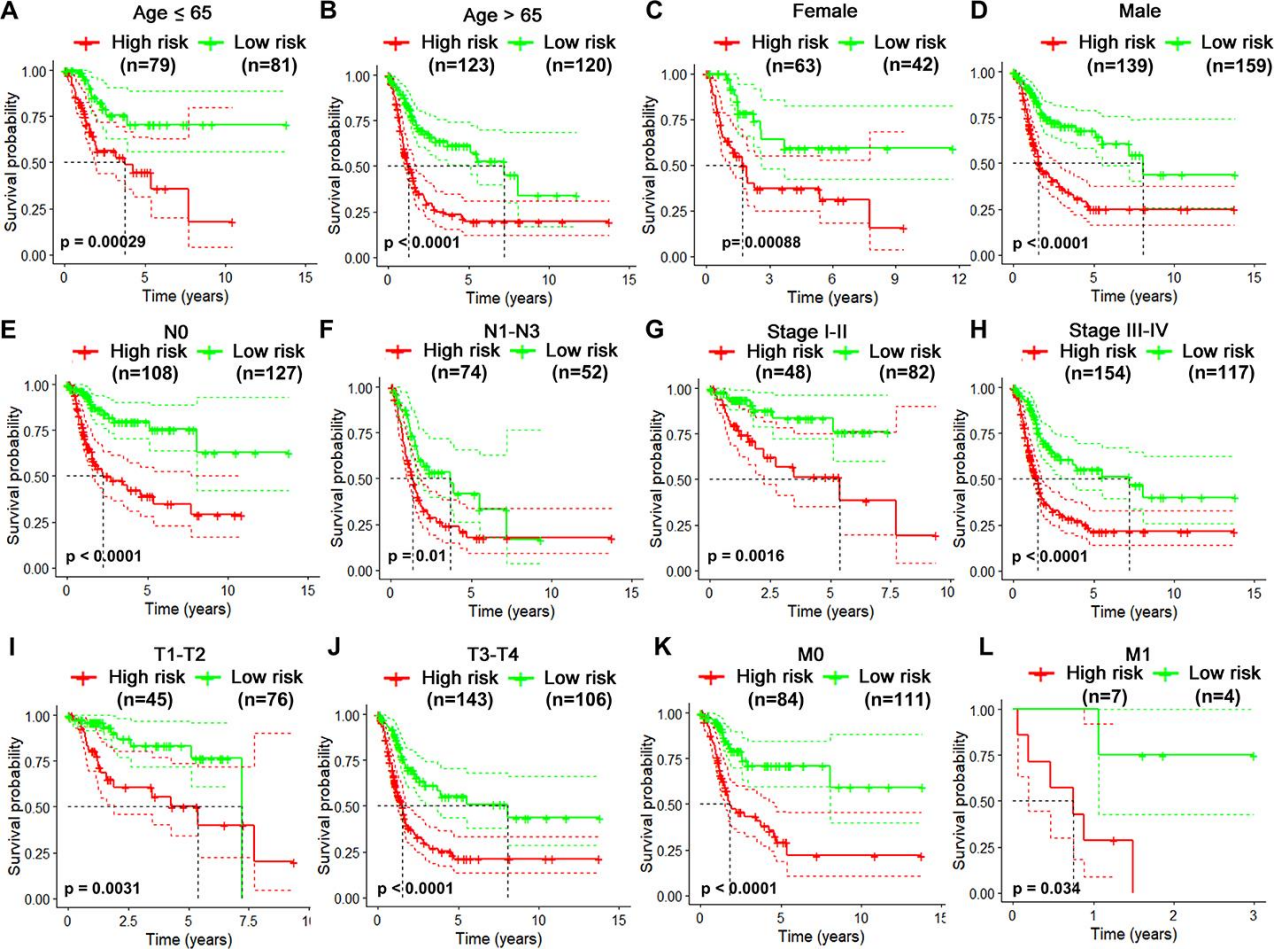
**Risk-stratified analysis of the 10-gene signature for overall survival of bladder cancer patients.** Kaplan-Meier curves for overall survival of patients in Age (≤65-year-old) subgroup (**A**), Age (>65-year-old) group (**B**), Female subgroup (**C**), Male subgroup (**D**), N0 subgroup (**E**), N1-N3 subgroup (**F**), clinical stage I-II subgroup (**G**), clinical stage III-IV subgroup (**H**), T1-2 subgroup (**I**), T3-4 subgroup (**J**), M0 subgroup (**K**), M1 subgroup (**L**).

Considering different clinical characteristics of bladder cancer patients, we performed a stratified analysis and subgroup analysis was stratified by age, gender, N stage, pathological stage, T stage and metastatic status (Figure 6A–6L). As shown in the Kaplan-Meier curves, we found that the survival rates of the high-risk groups had significantly decreased than the low-risk groups in any group. Thus, the predict power of overall survival by the 10-gene risk model was unrestricted to specific subgroups.

### Prognosis analysis and predictive accuracy of the 10-gene risk signature

**Figure 7:**
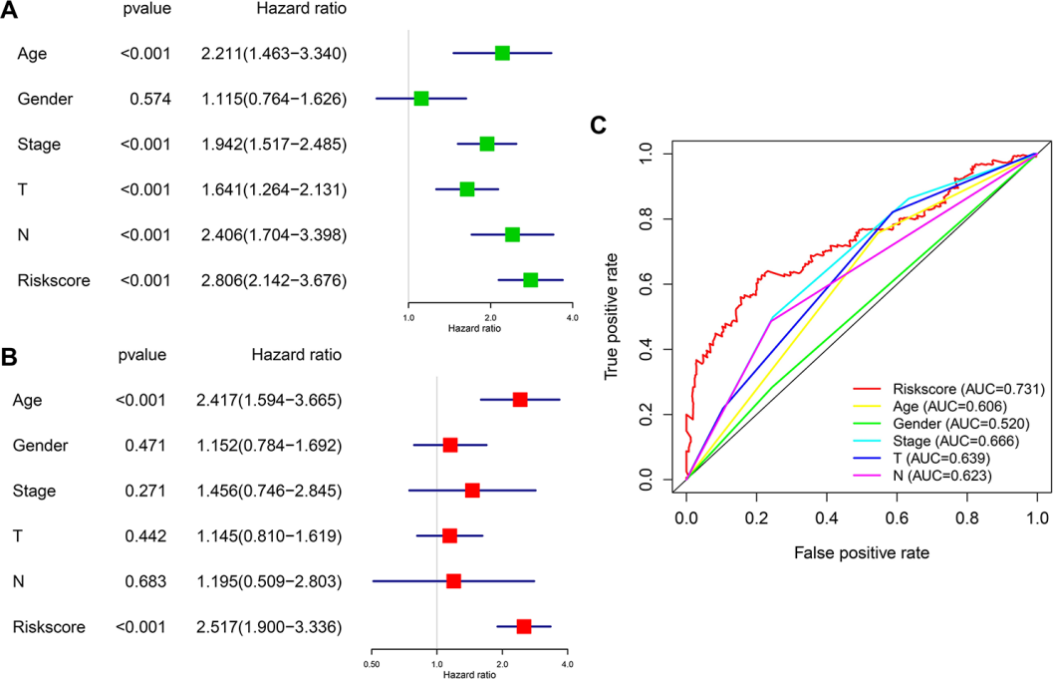
**Prognostic significance and predictive accuracy of the immune-related risk model.** (**A**, **B**) Univariate (**A**) and multivariate (**B**) Cox regression analyses of the risk scores of the clinical features for overall survival in TCGA-BLCA dataset. (**C**) ROC curve showing the specificity and sensitivity of the 10-gene signature risk score, age, gender clinical stage, T stage and lymph nodes status in predicting the OS of all UBC patients in the TCGA-BLCA dataset.

We then analyzed the relationship between OS, clinicopathological parameters, including gender, age, clinical stage, pathological features (T and N status), and risk score of the immune-related risk model in the TCGA cohort. Univariate Cox regression analysis revealed that the above clinical variables correlate with UBC survival, except gender and risk score (p<0.05; Figure 7A). Multivariable Cox regression analysis showed that this risk model acts as an independent prognostic indicator in the TCGA (Figure 7B). Next, ROC analysis was done to evaluate the risk signature specificity and sensitivity in predicting OS relative to clinicopathological parameters. Interestingly, risk score of the 10 genes exhibited better AUC relative the clinicopathological parameters (Figure 7C), indicating that the risk model independently predicts UBC survival. In the c-index analysis (Table 3), the risk model showed better predictive ability than that of the TNM stage in the training, validation, and entire cohorts.

**Table 3:** Harrell's concordance indexes of the risk score and stage in different cohorts.

**Cohort**	**Risk score**	**TNM Stage**
TCGA-Training	0.75 (0.72-0.78)	0.67 (0.61-0.74)
TCGA-Validation	0.67 (0.63-0.71)	0.62 (0.58-0.66)
TCGA-Entire	0.73 (0.69-0.75)	0.65 (0.63-0.67)
GSE13507-Validation	0.66 (0.62-0.70)	0.59 (0.55-0.63)

### Nomogram analysis of predictive accuracy

**Figure 8:**
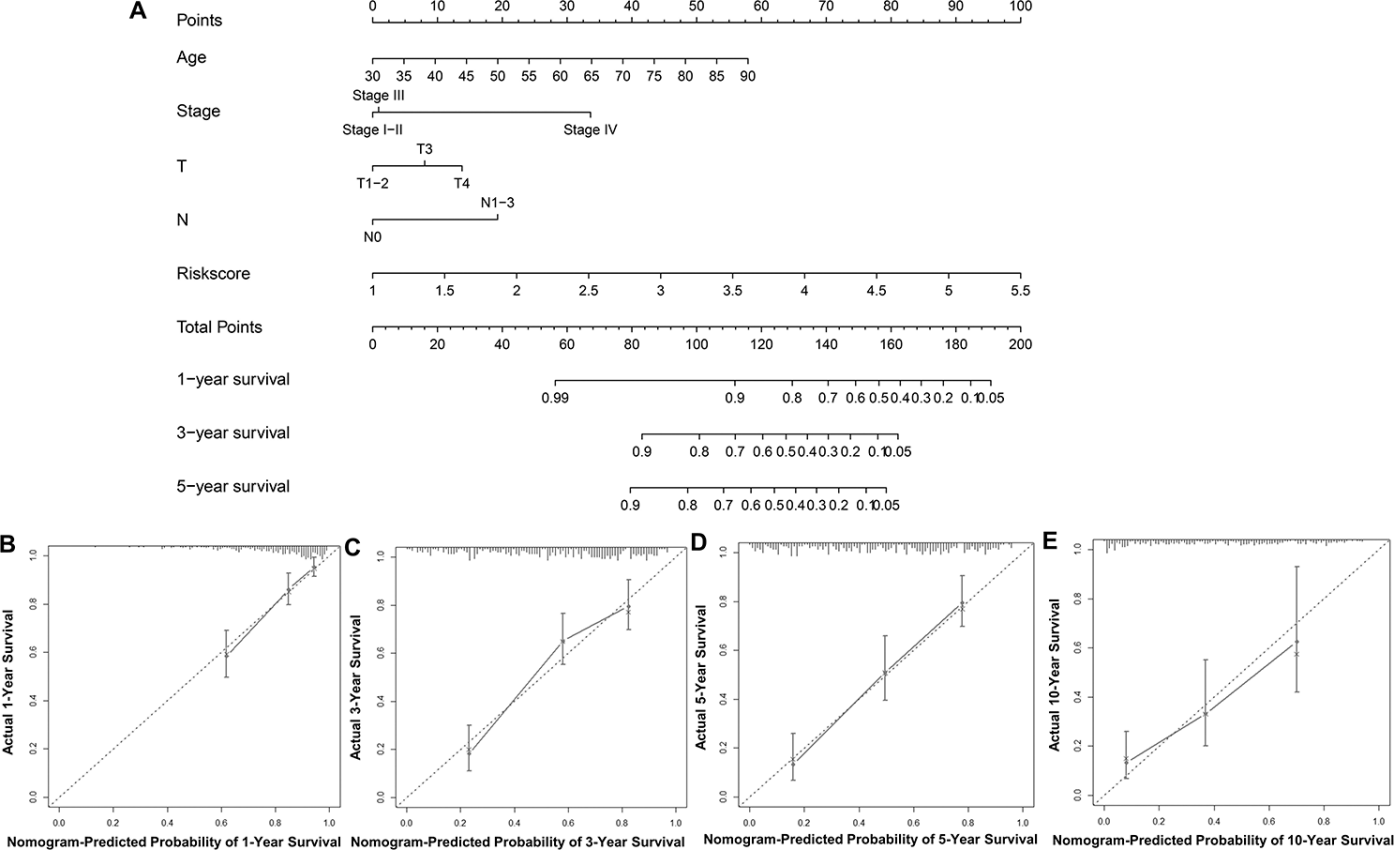
**Construction and validation of a nomogram.** (**A**) Nomogram for predicting the 1, 3, 5, and 10-year survival of UBC patients in the TCGA-BLCA cohort. (**B**–**E**) Calibration plot evaluating the consistency of the predicted value of the model and the probability of survival at 1, 3, 5, and 10 years obtained using the aforementioned nomogram.

To predict UBC survival in the TCGA cohort, we constructed a nomogram by integrating risk scores of the immune-related signature and clinical information, including age, tumor stage, T stage and N stage. On the nomogram, the score on the point scale is easily obtained for each variable. Thus, survival can conveniently be estimated at 1, 3, 5 and 10 years by calculating the overall points (Figure 8A). The nomogram’s accuracy was evaluated using calibration curve analysis, which revealed consistency between the predictive curves at 1, 3, 5 and 10 years, and the ideal curve (Figure 8B–8E), indicating excellent performance by our nomogram. These data suggest that the nomogram is an effective tool for predicting UBC survival.

### Correlation of tumor-infiltrating immune cells (TIICs) with the 10-IRG risk model

**Figure 9:**
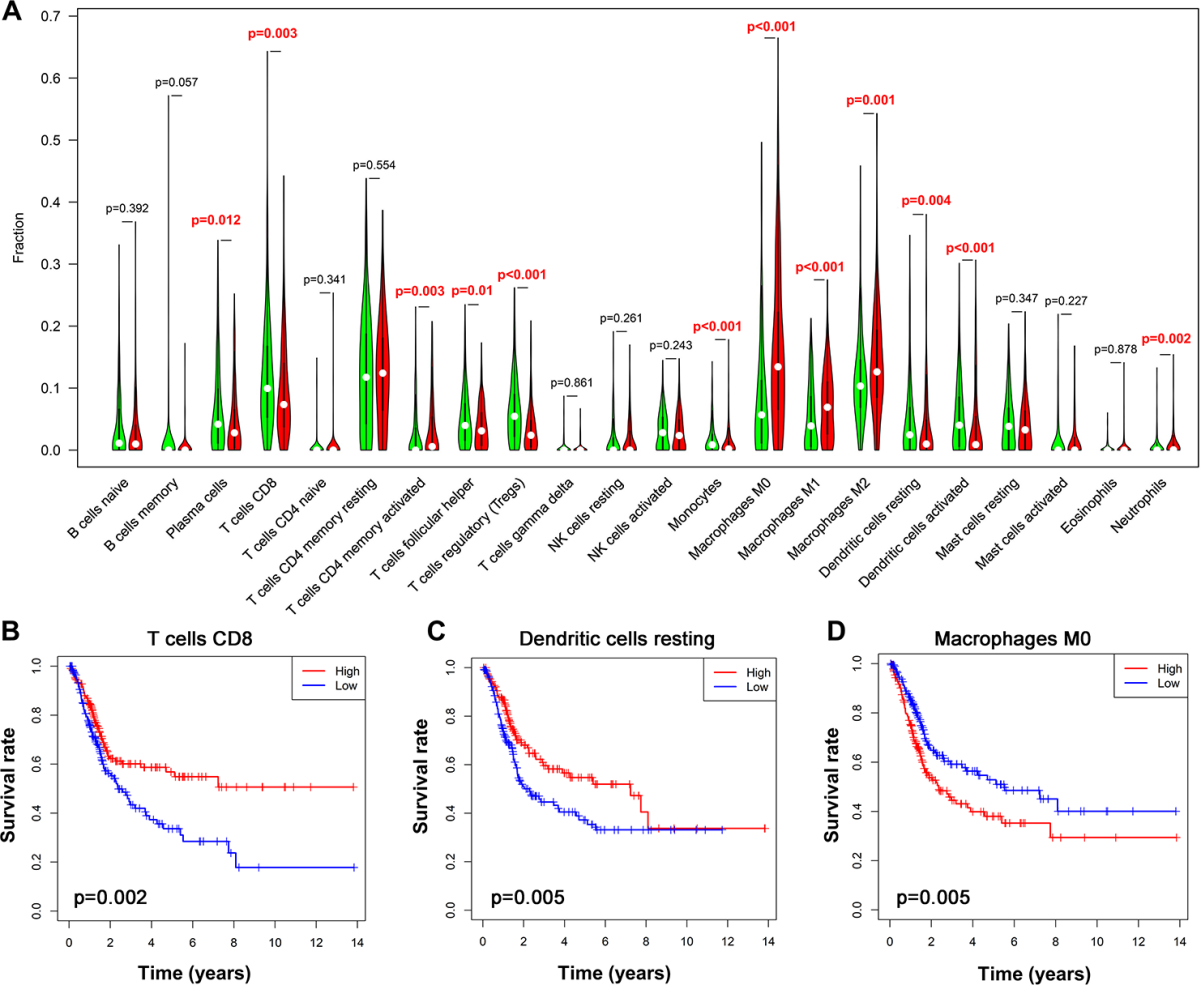
**Alteration of immune cell infiltration in BLCA samples with different risk status.** (**A**) Violin plot demonstrating the TILCs associated with the risk model. High- and low-risk groups are represented by red and green violin, respectively. (**B**–**D**) Kaplan-Meier curve analysis of overall survival for various immune cells infiltration. (**B**) CD8 T cells; (**C**) dendritic cells resting; (**D**) macrophages M0.

TIICs have been suggested as independent predictors of survival in various cancer and are regulated by immune-related genes [27, 28]. We therefore evaluated the correlation between immune cells fractions in UBC tissues and risk models using CIBERSORT [29]. This analysis revealed that among the 22 immune cells, activated CD4+ memory T cell, macrophages and neutrophils positively correlated with the 10-IRG risk status, while CD8+ T cells, follicular helper T cells, regulatory T cells, and monocytes negatively correlate with the immune-related risk score (Figure 9A). To cross examine the data, further analysis showed that macrophages and neutrophils were positively correlated with the 10-IRG risk status, while CD8+ T cells were negatively correlate with the immune-related risk score with both methods for deconvolution (Figure 9A and Supplementary Figure 3). Moreover, a correlation heatmap demonstrated that different immune cells fractions have a weak/moderate correlation (Supplementary Figure 4). The proportion of CD8+ T cells, dendritic cells resting, and macrophages (Figure 9B–9D) significantly correlate with survival. These data may reveal the poor clinical outcome in the high-risk cohort in part.

### Relationship of immune-related risk model with mutation profile, molecular subtype and immunotherapy markers

**Figure 10:**
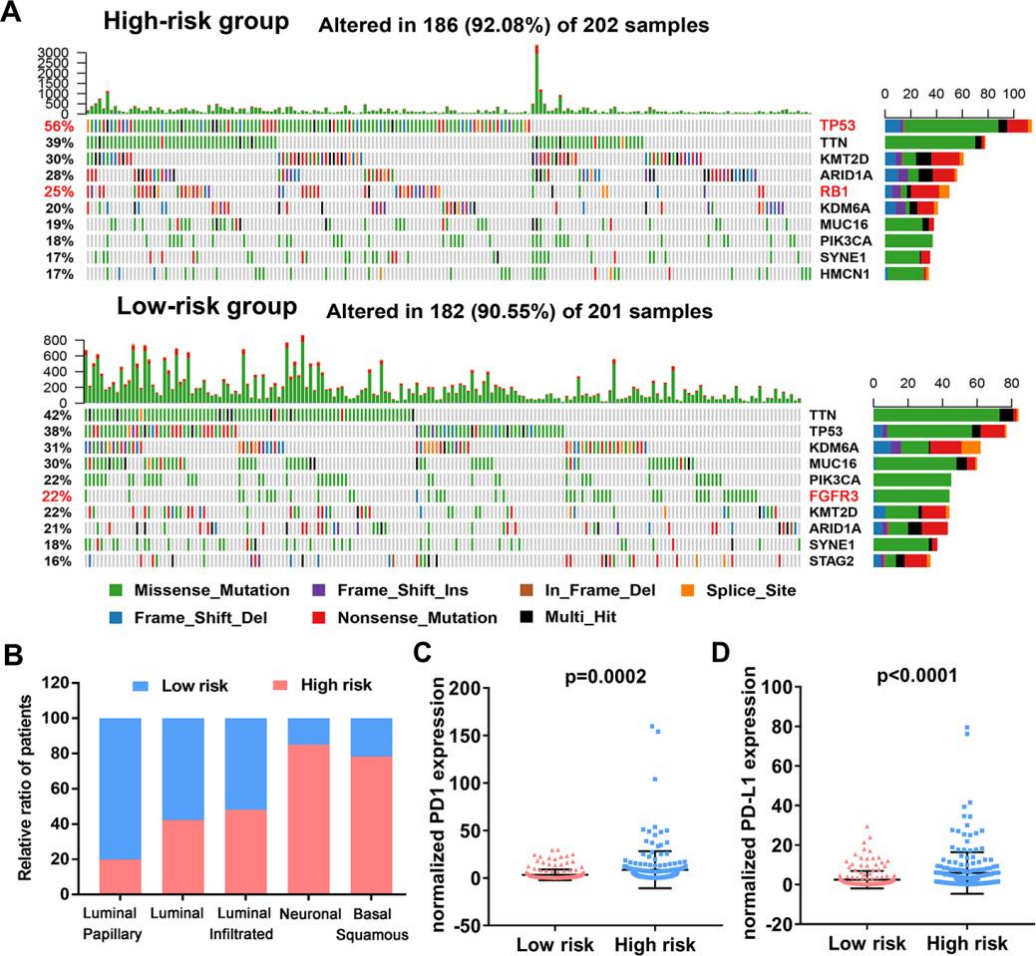
**Variations in the mutation landscape and molecular features of the high and low-risk UBCs in TCGA-BLCA cohort.** (**A**) Mutation landscapes of UBC samples in the low- and high-risk groups. (**B**) Association between the 10-genes risk model and molecular subtypes of UBC patients in the TCGA-BLCA dataset. (**C**, **D**) Normalized PD1 and PD-L1 gene expression profile in the low- and high-risk groups.

To assess if the immune-related risk signature was associated with specific tumor mutations, the correlation between the signature and mutations was analyzed in TCGA BLCA dataset containing somatic mutation profiles. The overall landscape of mutation profile was illustrated in Supplementary Figure 5**.** Alterations in the mutation landscape in high or low risk group were as follows: 6 genes were mutated in >19% of tissues with high risk score: *TP53* (56%), *TTN* (39%), *KMT2D* (30%), *ARID1A* (28%), *RB1* (25%) and *KDM6A* (20%). While eight genes were mutated in >19% of tissues with low risk score: *TTN* (42%), *TP53* (38%), *KDM6A* (31%), *MUC16* (30%), *PIK3CA* (22%), *FGFR3* (22%), *KMT2D* (28%) and *ARID1A* (21%) (Figure 10A). Notably, higher rates of FGFR3 mutation occurred in bladder cancer tissues with low risk score compared with tumors with high risk score. However, high TP53 and RB1 mutation rates occurred in the high-risk subgroup. Next, we evaluated the relationship between the risk signature and UBC molecular subtypes. Interestingly, for luminal papillary UBC, strikingly more patients had low risk scores relative to those with high risk scores. For basal-squamous and neuronal UBC most patients had high risk scores (Figure 10B). Moreover, patients with high risk exhibited higher *PD1* and *PD-L1* expression (*p*<0.05; Figure 10C, 10D). These results suggest that high-risk patients in the 10-IRG signature are promising candidates for immune checkpoint inhibitors or other treatment management.

### Identification of ten-gene risk score correlated biological pathways

**Figure 11:**
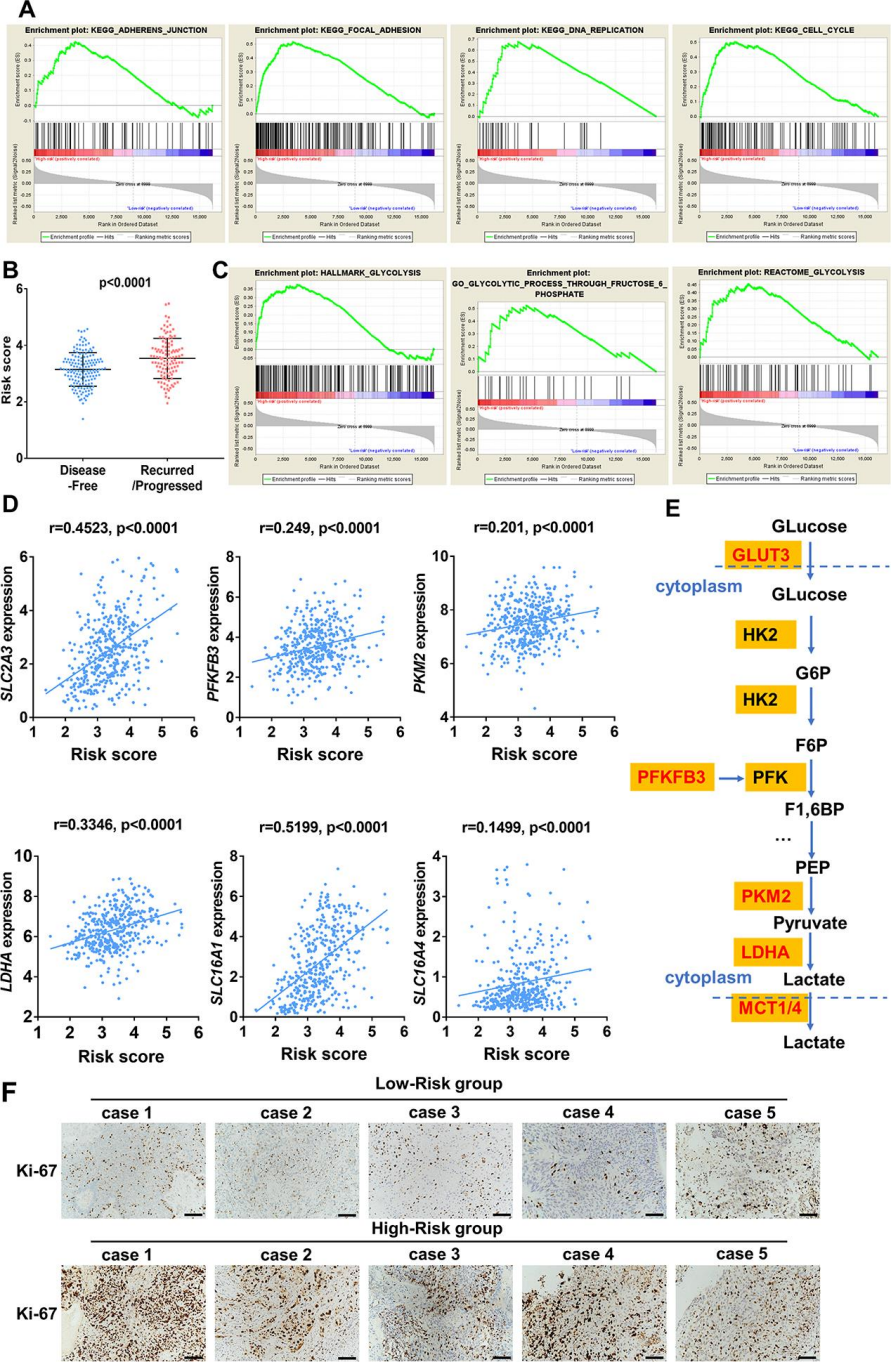
**Enrichment analysis of cancer and glycolysis related pathways associated with risk score in TCGA-BLCA database.** (**A**) Gene set enrichment analysis (GSEA) on the association of risk score with adherens junction, focal adhesion, cell cycle and DNA replication. (**B**) Risk score of UBC patients with disease free and progression/recurrence in TCGA-BLCA dataset. (**C**) GSEA plots depicting enrichment of glycolysis pathways and risk score. (**D**) The correlation between the expression of glycolytic genes and the risk score. (**E**) The identified core candidate genes (red) involved in enzymatic glycolysis are shown. (**F**) The representative IHC images of ki-67 expression in UBC tissues with low- or high-risk.

In order to investigate biological function of high- and low risk score in bladder cancer, GSEA was performed and unsurprisingly, the results revealed that high-risk score was closely correlated with cancer related pathways including ADHERENS JUNCTION, FOCOL ADHESION, CELL CYCLE, DNA REPLICATION (Figure 11A). Moreover, patients with recurrence/ progression tended to have a higher risk score than those with disease free (Figure 11B). Unexpectedly, genes involved in glycolysis were significantly enriched in patients with a high-risk score (Figure 11C). Significant positive correlation between core glycolytic genes, including *SLC2A3*(GLUT3), *PFKFB3*, *PKM2*, *LDHA* and *SLC16A1/4*(MCT1/4), and risk score was observed (Figure 11D, 11E). These results indicate that this ten-gene risk score is closely related to tumor progression and glycolytic status of UBC. Interestingly, we observed a trend for higher expression of Ki-67 in high-risk groups versus the low-risk group (Figure 11F).

### Regulatory network among differentially expressed TFs and IRGs

**Figure 12:**
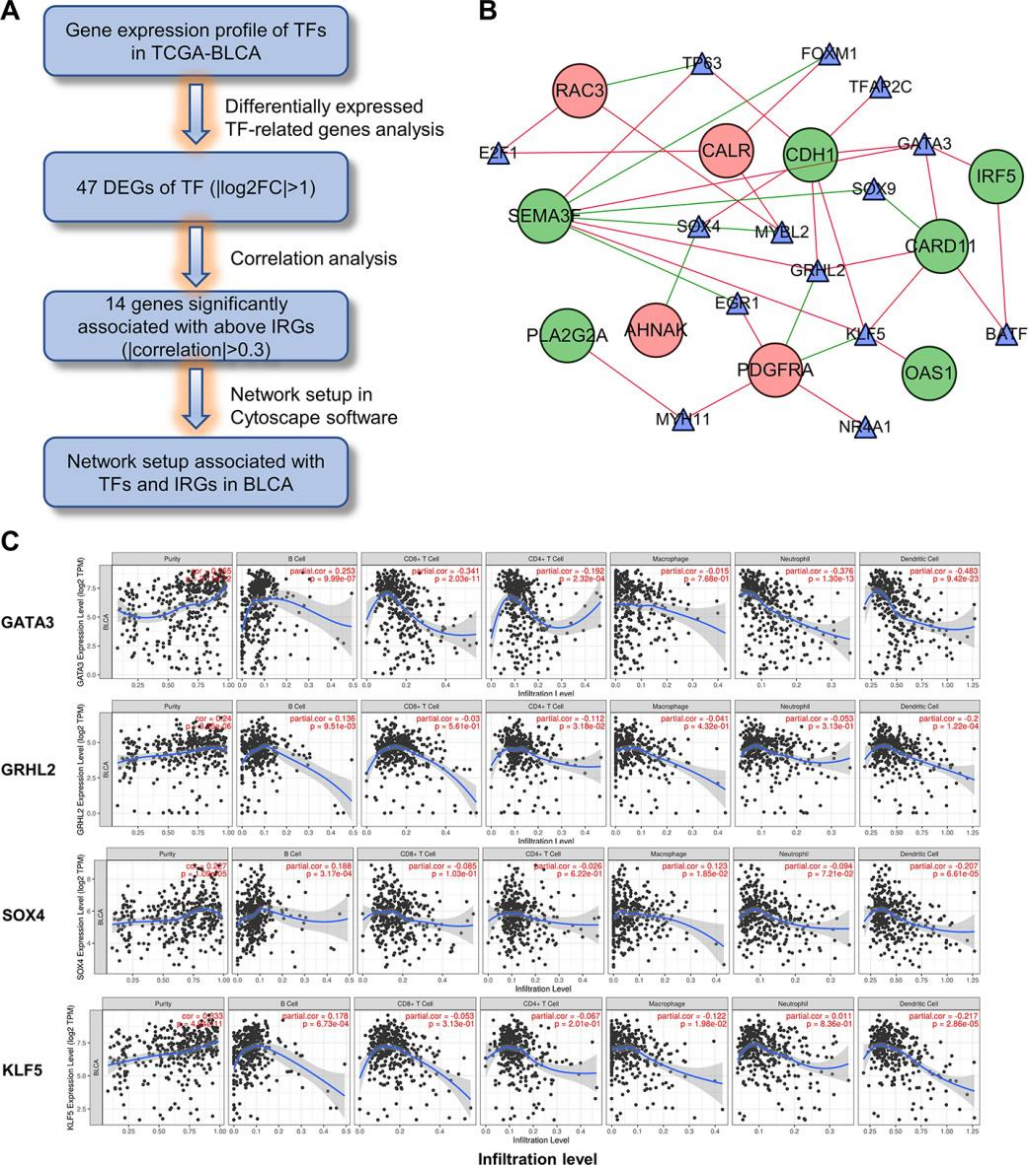
**Construction of the TF-IRG network in UBC group.** (**A**) Diagram showing the construction of regulatory network of TFs and genes in the immune-related risk signature. (**B**) Regulatory network of the differentially expressed TFs and IRGs. (**C**) Correlation of GATA3, GRHL2, SOX4 and KLF5 expression with immune cell infiltration level by TIMER in UBCs.

To elucidate the mechanisms involved in the regulation of these immune-related genes, we examined differentially expressed transcription factors and correlation analysis of DEGs between the 2 gene lists (Figure 12A). Construction of the regulatory network revealed *KLF5*, *SOX4*, *GRHL2* and *GATA3* as the nodes with highest connectivity (Figure 12B). Interestingly, further investigation via Tumor Immune Estimation Resource (TIMER) showed these transcriptional factors, particularly GATA3, were closely related to the immune cell infiltration of tumors (Figure 12C). These results indicate that the 4 genes may involve in the upstream regulation of the ten IRGs and influences the immune status of the tumor microenvironment.

## DISCUSSION

Mounting evidence has shown that not all patients benefit from chemotherapy [30]. However, despite growing interest in immunotherapy, only a small proportion UBC patients respond to immunotherapy [31]. Therefore, personalized therapies are proposed as means to optimize outcomes. The complexity of the response to immunotherapy makes it impossible that a single gene could be sufficient to predict prognosis and response to immunotherapy. Recent studies have reported multiple gene expression signatures, including immune-related signature, that can predict cancer outcomes patients with UBC [21, 25]. However, very few of these are prognostic or capable of guiding treatment. Here, we have identified an immune-related risk model for UBC by analyzing TCGA and GTEx datasets. Using the 2 datasets minimizes bias from having too few normal samples in the TCGA dataset.

The risk model comprises 10 differentially expressed IRGs (DEIRGs) that correlate with prognosis. We demonstrate that the risk model based on the immune-related genes is an accurate predictor of UBC survival by validating it in 2 independent UBC datasets. Multivariate Cox regression analysis revealed that the risk score is an independent predictor for UBC prognosis. Our study also reveals correlation between the risk model and several clinicopathological factors including invasive status, clinical stage, and T and N stage. Nomogram analysis using risk score, clinical characteristics and patient information revealed a high predictive performance by the risk model. These results indicate that the signature can serve as an accurate prognostic tool.

Among these risk genes, 5 DEIRGs (*AHNAK*, *CALR*, *PDGFRA*, *RAC3*, *PLA2G2A*) correlated with high risk and 5 (*OAS1*, *CDH1*, *SEMA3F*, *IRF5*, *CARD11*) were protective factors. AHNAK1 is a scaffold protein, highly expressed by CD4+ T cells, and is a critical component for calcium signaling. AHNAK1 deficiency resulted in a reduced calcium influx upon TCR crosslinking [32]. Calreticulin (CALR) is a Ca2+-binding endoplasmic reticulum (ER) protein that contributes to the initiation of adaptive anticancer immunity in the context of immunogenic cell death (ICD), an immune response that eradicates cells experiencing damage beyond recovery in support of organismal fitness [33]. Phospholipase A2 group IIA (PLA2G2A) is an antimicrobial molecular which shows bactericidal activity against bacteria. Upregulation of PLA2G2A in cancer cell significantly suppressed L. monocytogenes infection. It has been suggested that the anti-bacterial activity of Pla2g2a is involved in tumor inhibition [34]. OAS1 is a member of OAS family which acts as antiviral enzymes induced by interferon. It was reported that neutrophils, which contribute to tumor metastasis through multiple pathways, were the top tumor immune infiltrating cell type associated with OAS in in breast cancer [35]. CDH1 deletions have been shown to cause cancers with immune cell infiltration, activation of targetable immune checkpoint pathways and gene expression related to T-regulatory (Treg) cell signaling [36]. Platelet-derived growth factor receptor alpha (PDGFRA) belongs to the type III receptor tyrosine kinase family. It has been reported that imatinib downregulates PD-L1 and IRF1 expression through the inhibition of KIT and PDGFRA, thus contributing to counteract the suppressed adaptive immune response against GIST [37]. SEMA3F is a member of Semaphorins and is found to regulate immune cell trafficking during the development of thymocytes, and it is also found to regulate NK-cell migration and NK–DC interactions [38]. RAC3 was reported to show antitumor activity through maintaining the maturation and effector function of NK cells via modulation of several critical T-bet-dependent genes such as IRF5 [39]. Transcription factor interferon regulatory factor 5 (IRF5) has been shown to regulate the expression of genes involved in the stimulation of the immune system. The GM-CSF-IRF5 signaling axis has been reported to promote antitumor immunity through activation of CD8 T cell infiltrates [40]. CARD11-BCL10-MALT1 (CBM) signaling mediates TCR-induced NF-κB activation in Tregs and controls the conversion of resting Tregs to effector Tregs and disruption of the CBM complex is sufficient to prime the tumor environment for successful immune checkpoint therapy [41]. *AHNAK* was identified as a novel candidate biomarker [42] for UBC diagnosis by liquid-based cytology and its prognostic value was demonstrated in other studies [43]. Somatic *CDH1* mutations are frequent in UBC and promote UBC progression [44]. *CDH1* deletions have been shown to cause cancers with immune cell infiltration, activation of targetable immune checkpoint pathways and gene expression related to T-regulatory (Treg) cell signaling, suggesting immune checkpoint potential therapeutic options [36]. Although the remaining 8 genes have not been associated with UBC, their roles and their prognostic value in UBC merit investigation.

Given that multiple genes mentioned above modulate the tumor microenvironment in cancers and that TIICs may contribute to UBC progression [45, 46], we explored the proportions of TIICs in UBC and the correlation of risk status with various immune cell subtype. The proportion of CD8+ T cells, Tregs, and dendritic cells were lower in the high-risk group according to the 10-gene risk model. High CD8+ T cells and dendritic cells proportions correlated with better survival. In contrast, the proportion of macrophages and neutrophils were high in the high-risk set, in which the high proportion of macrophages indicated poorer OS. Moreover, high CD8+ T cells and macrophage infiltration correlated with longer and shorter UBC survival, respectively, which is consistent with previous studies [47, 48]. Although neutrophils do not correlate with OS, they have been reported to correlate with negative prognostic in some cancer types [49]. Nonetheless, the function of Tregs and monocytes in UBC prognosis remains controversial. Changes in tumor immune cells may partly explain the prognostic value of this model.

To investigate the mechanisms underlying this risk model, we analyzed the UBC mutation profiles. Interestingly, we uncovered a subgroup with low-risk scores that featured higher FGFR3 mutation rates. Moreover, for the luminal papillary subtype most patients have low risk scores [5]. The luminal papillary subtype cohort was characterized by papillary morphology, lower stage, lower risk for progression and enrichment with *FGFR3* mutations. These results suggest tyrosine kinase inhibitors against *FGFR3* as a therapeutic option in patients with low risk scores. Patients with high risk score accounted for the bulk of basal-squamous and neuronal subtypes, which had high rates of *TP53* and *RB1* mutations, suggesting the worse survival, making it significant to recognize clinically. In addition, high levels of immune markers and mesenchymal expression signature in basal-squamous subtype suggested resistance to cisplatin-based chemotherapy, and sensitivity to immune checkpoint inhibition. However, despite high PD1/L1 expression in high-risk tumors, glycolytic pathways also simultaneously enriched. Cancer cells are known to reprogram their metabolism by upregulating glucose uptake. Enhanced glycolysis within the TME results in an accumulation of lactic acid [50] and glucose-deprived niche can directly inhibit glycolysis in immune cells, thus hindering antitumor immune responses. Taken together, these results suggest that the immune-related risk signature may help personalize therapies. Glycolysis inhibition coupled with immunotherapy design may be a rational approach to improve the efficacy of ICIs.

TFs bind to promoter regions of specific genes to modulate their expression. To identify the modulators of these immune-related genes, we constructed a regulatory network of DEGs between the TF and IRG genes. This analysis revealed *KLF5*, *SOX4*, *GRHL2* and *GATA3* as nodes with highest connectivity, suggesting them as core genes regulators. *KLF5* is a known oncogene in bladder cancer that promotes angiogenesis and invasion [43]. Low *GATA3* levels have been reported to regulate self-renewal and tumorigenicity in bladder cancer stem cells by modulating STAT3 expression [51].

A limitation of this study is its reliance on public datasets, whose accompanying information is limited. Thus, these findings need to be validated in future clinical studies. Moreover, the proportion of Asian patients in our study was small. Future studies should incorporate more Asian UBC samples.

In conclusion, we have developed and validated a reliable 10-IRG signature model for predicting UBC patient outcomes and potential therapeutic options. Our data suggest that changes in mutation landscape, TIME and glycolytic status might be probable causes for this signature’s prognostic capacity. These findings may unveil novel, potential immune biomarkers and therapeutic strategies. Additional studies are needed to confirm the prognostic value of this signature.

## MATERIALS AND METHODS

### Patient data and databases

A comprehensive list of IRGs was obtained from Immunology Database and Analysis Portal (ImmPort) database (https://immport.niaid.nih.gov) [51]. UCSC database (https://xena.ucsc.edu/) was utilized to obtain gene expression data of normal bladder samples from the GTEx database. Data on normal and UBC tissues was downloaded from the Cancer Genome Atlas (TCGA) data portal (https://portal.gdc.cancer.gov/). To get a unified form, the RNA-seq data in the 2 databases were converted into the log2(x+1) form and then normalized. Clinical data was downloaded and organized from TCGA. The gene expression data and corresponding clinical information of microarray dataset from GSE13507, which used the Illumina human-6 v2.0 expression beadchip, were downloaded from Gene Expression Omnibus (GEO) (https://www.ncbi.nlm.nih.gov/geo/).

### Differential gene analysis

Analysis of differential gene expression between normal and UBC tissues was done using the Wilcoxon signed-rank test, applying FDR <0.05 and |log2fold change (FC)|>1 as cutoff threshold. Intersection between the DEG list and IRG list or TF list was established to identify differentially expressed immune-related genes (DEIRGs).

### Functional enrichment analysis

In order to explore the underlying molecular mechanisms of DEGs, GO term and KEGG pathway analysis were done using clusterProfiler R package and were visualized using “ggplot2” [52] on R. Protein-protein interaction enrichment analysis was done on Metascape (http://www.home-for-researchers.com) [53] using OmniPath8, BioGrid6 and InWeb_IM7. Closely connected network components were identified using the Molecular Complex Detection (MCODE) [54].

### Construction and validation of an immune-related prognostic model

UBC cases in the TCGA dataset that had overall survivals >0 days were randomly classified into a training and a testing group. The training group was used to identify the prognostic DEIRGs using univariate Cox regression analysis (p≤0.05), to establish a prognostic immune-related risk model using Least Absolute Shrinkage and Selection Operator (LASSO) regression analysis to minimize overfitting, and to seek out the optimal gene pattern using glmnet on R [55]. Risk scores for each patient in the training set and testing set were calculated using the formula: Risk score = exprgene1* coefficient gene 1+exprgene 2 * coefficient gene 2+ ⋯ + exprgene10 * coefficient gene 10. UBC cases in the training group were grouped into high and low-risk categories based on the median risk score. The signature’s prognostic value was tested on the TCGA and GSE13507 cohorts. To validate the prognostic power of the IRG risk model, area under the curve (AUC) was calculated with the timeROC R package. The discrimination of the risk models was measured and compared by Harrell’s concordance index (c-index). Survival analysis was performed using the survminer R package and visualized using ggplot2 R package.

### TF-IRG network construction

To explore potential relationships between TF and IRGs, correlation analysis of DEGs between the 2 gene lists was carried out**.** Significant results in correlation analysis were used to construct a regulatory network, which was visualized using Cytoscape. Correlation values between TF and IRGs >0.3 were considered significantly correlated. Based on the co-expression network, we analyzed the regulatory relationship of genes and identified core regulatory TF genes.

### Estimate of tumor-infiltrating immune cells

RNA-seq gene expression data on bladder cancer dataset (TCGA-BLCA) from TCGA database were used to estimate the relative proportion of infiltrated immune cells utilizing the CIBERSORT R package and TIMER [29, 56].

### Mutation analysis

Mutation annotation format (MAF) containing somatic variants data was downloaded from the TCGA database. MAF files were then visualized and summarized from this study using the maftools Bioconductor package [57].

### Statistical analysis

Continuous variables were described as mean ±S.D., while categorical variables were presented by frequency (n) and proportion (%). Statistical analysis was performed using the R software or GraphPad Prism version 6.0. p<0.05 was considered statistically significant. Univariate and multivariate analyses were performed using the Cox proportional hazards regression model to evaluate the prognostic effect of our immune-related signature and other clinicopathological features. Time-dependent ROC analysis was utilized to assess the accuracy of the immune-related prognostic model. The log-rank test in Kaplan–Meier survival curves was performed to analyze differences in overall survival (OS).

## Supplementary Material

Supplementary Figures
