# Supplementary material for: Identification of a novel immune microenvironment signature predicting survival and therapeutic options for bladder cancer
Source: Aging (Albany NY). 2020 Dec 19;13(2):2780–802. doi: 10.18632/aging.202327 (PMC7880321; doi:10.18632/aging.202327)
Supplement: Supplementary Figures [file aging-13-202327-s001.pdf]

SUPPLEMENTARY FIGURES

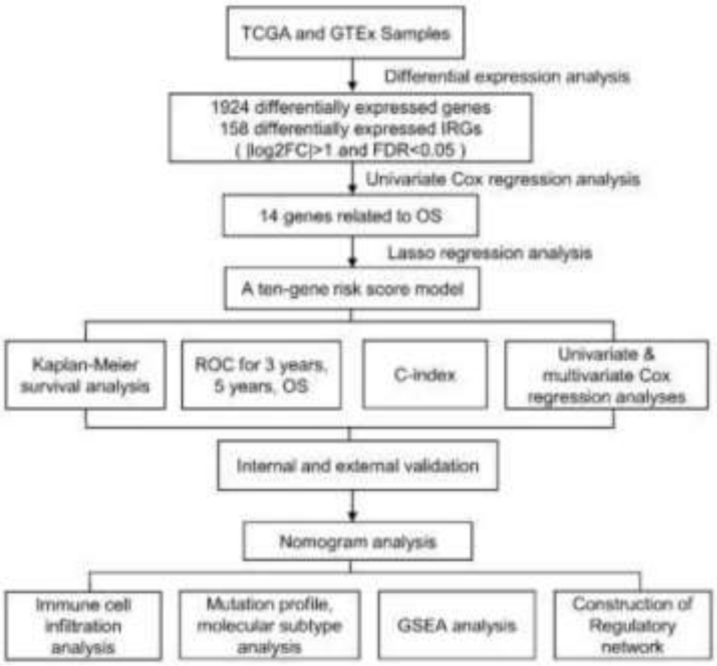

Supplementary Figure 1. An overall workflow describing the process used to develop and validate the prognostic model to predict prognostic outcomes.

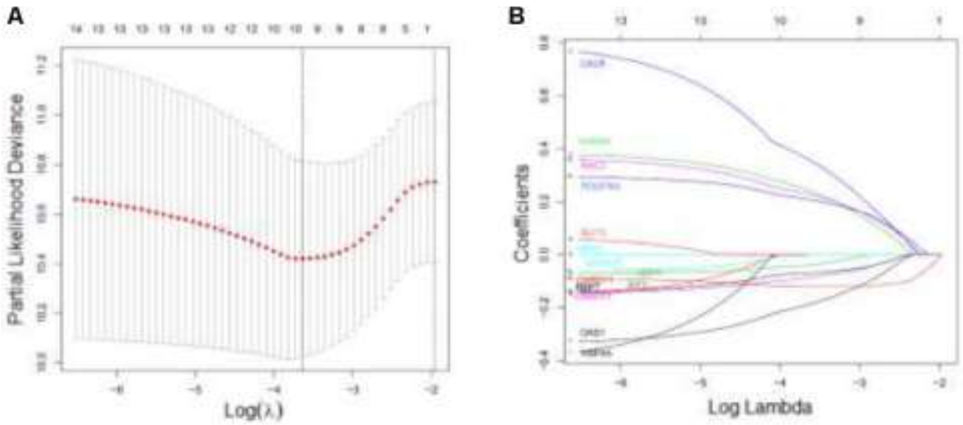

Supplementary Figure 2. Lasso regression analysis of IRGs with prognostic potential in the training cohort of UBC patients. (**A**, **B**) Lasso regression analyses of DEIRGs using the OS model. The optimal values of the penalty parameter λ were determined by cross-validation.

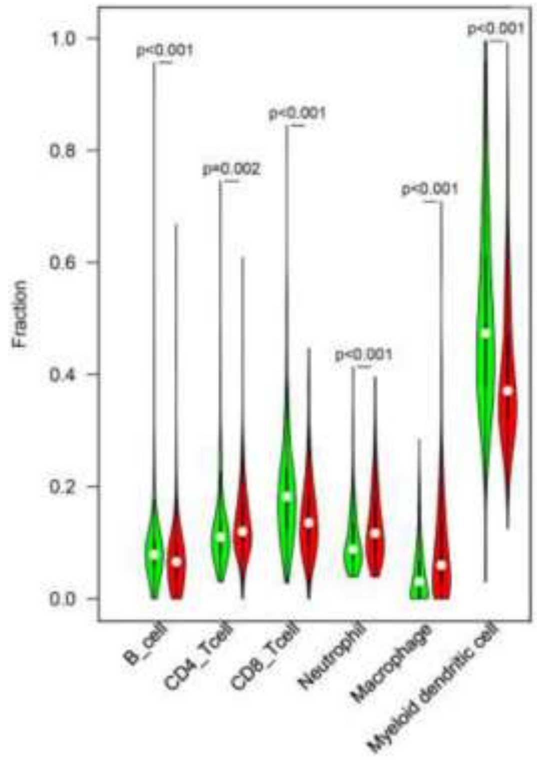

Supplementary Figure 3. Violin plot demonstrating the TILCs associated with the risk model by TIMER. High- and low-risk groups are represented by red and green violin, respectively.

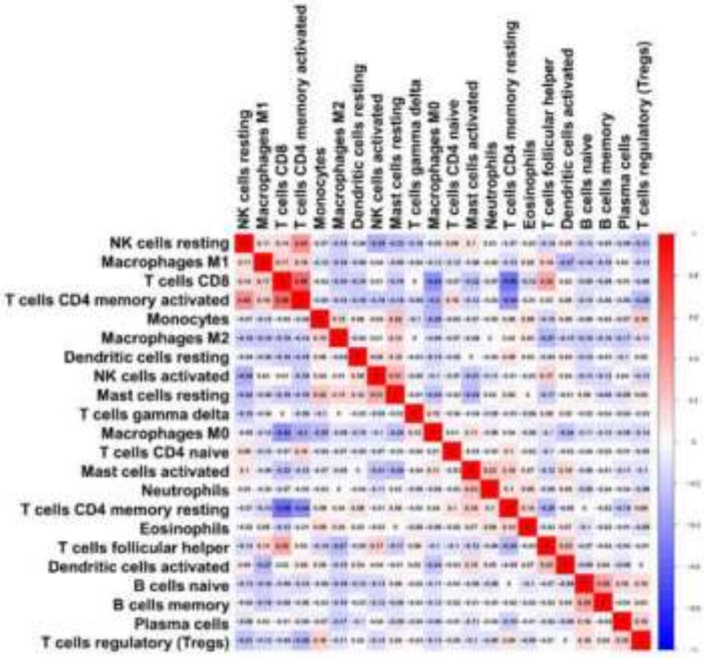

Supplementary Figure 4. Correlation heatmap showing the proportions of different TIICs clusters.

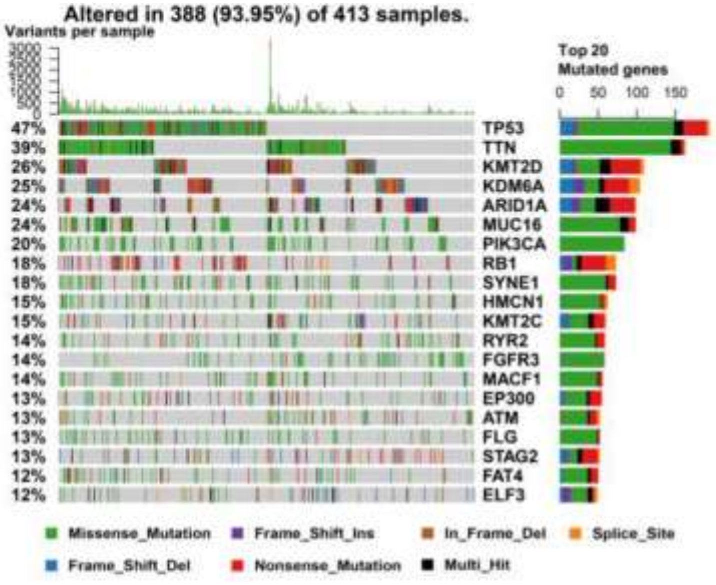

Supplementary Figure 5. Landscape of mutation profile in TCGA-BLCA dataset.
